# Canine separation-related behaviour at six months of age: Dog, owner and early-life risk factors identified using the ‘Generation Pup’ longitudinal study

**DOI:** 10.1017/awf.2024.56

**Published:** 2024-12-16

**Authors:** Fiona C. Dale, Charlotte C. Burn, Jane Murray, Rachel Casey

**Affiliations:** 1 Animal Welfare Science and Ethics, Royal Veterinary College, North Mymms AL9 7TA, UK; 2 Dogs Trust, London EC1V 7RQ, UK

**Keywords:** animal welfare, anxiety, dogs, early experience, human-animal interactions, separation-related behaviour

## Abstract

Separation-related behaviours (SRBs) in dogs (*Canis familiaris*) often indicate poor welfare. Understanding SRB risk factors can aid prevention strategies. We investigated whether early-life experiences and dog-owner interactions affect SRB development. Using a longitudinal study, we conducted exploratory analyses of associations between potential risk factors and SRB occurrence in six month old puppies (n = 145). Dogs were less likely to develop SRBs if owners reported that, at ≤ 16 weeks old, puppies were restricted to crates/rooms overnight and had ≥ 9 h of sleep per night. Puppies with poor house-training at ≤ 16 weeks were more likely to show SRBs, as were those trained using dog treats or novel kibble versus other rewards. Puppies whose owners used more punishment/aversive techniques when responding to ‘bad’ behaviour had increased odds of SRBs at six months versus other puppies. Puppies whose owners reported ‘fussing’ over their dogs at six months in response to ‘bad’ behaviour upon their return, versus those whose owners responded in other ways, were six times more likely to display SRBs. Other factors, including dog breed, sex and source, showed no significant association with SRB occurrence. Thus, SRB development might be prevented by enabling sleep for ≥ 9 h in early life, providing enclosed space overnight, refraining from aversive training of puppies generally, and avoiding fussing over puppies in response to unwanted behaviour following separation. These recommendations derive from correlational longitudinal study results, so analysis of interventional data is required for confirmation regarding effective prevention strategies.

## Introduction

Canine separation-related behaviours (SRBs) can be associated with poor canine welfare, but the factors leading to their development are little understood, making prevention potentially difficult. These behaviours occur when owners are absent, typically starting within a few minutes of owner departure (Palestrini *et al.* 2010). The most commonly reported SRBs are destructive behaviours, such as chewing and scratching, often near the site of owner departure, and vocalisations such as barking, howling and whining (Lund & Jørgensen 1999). Dogs (*Canis familiaris*) may also urinate, defaecate, or display repetitive behaviours such as spinning or pacing (Blackwell *et al.* 2006; Palestrini *et al.* 2010). Behaviours relating to dogs being left alone are commonly reported as being problematic for dog owners and are often included as leading reasons for behavioural referrals (Bamberger & Houpt 2006; Storengen *et al.* 2014; Siracusa *et al.* 2017). There is a range of terminology used to describe separation behaviours in the literature (Ogata 2016; de Assis *et al.* 2020), including SRBs, separation anxiety, separation-related disorders, separation-related distress and separation-related problems (e.g. Takeuchi *et al.* 2001; Blackwell *et al.* 2006; Konok *et al.* 2011; de Assis *et al.* 2020). Here, we refer to problematic canine behaviours when owners are absent as SRBs, so as not to make assumptions regarding the severity of such behaviours or the underlying emotions, which could include frustration, fear, anxiety or boredom (McCrave 1991; Mendl *et al.* 2010a; Burn 2017; de Assis *et al.* 2020) or categories described in de Assis *et al.* (2020) such as panic and desire.

The prevalence of SRBs has been reported from 6 to 55% (Bradshaw *et al.* 2002; Soares *et al.* 2010; Blackwell *et al.* 2016; Dinwoodie *et al.* 2019; Salonen *et al.* 2020; Harvey *et al.* 2022; Brand *et al.* 2024). The wide range in prevalence estimates is likely to be partly due to the difficulty in observing behaviour that, by definition, occurs when no humans are present. Prevalence estimations from owner-report (e.g. Tiira *et al.* 2016; Dinwoodie *et al.* 2019; Salonen *et al.* 2020) are often based on what owners could hear upon their approach to the household immediately prior to their return, neighbour reports, or visible damage to the household or other items. Therefore, SRBs displayed during owner absence, without witness or evidence, may be missed from owner-report which may mean prevalence measured in this way is underestimated. Furthermore, subtle SRBs observable via video, such as panting, lip-licking (Palestrini *et al.* 2010), salivation, repetitive behaviour (Blackwell *et al.* 2006), or depression-like behaviours (Podberscek *et al.* 1999; Landsberg *et al.* 2008; Soares *et al.* 2010), may go unnoticed by owners or may not be attributed to problems with being left alone. The prevalence of SRBs may therefore be higher than reported, meaning more dogs than estimated may be experiencing SRBs, potentially on a daily basis, when their owners are absent.

Risk factors for SRB development have been researched to some extent, but almost exclusively in adult dogs rather than in puppies. Risk factors can include characteristics of the dogs themselves, their owners, or their environment and early experiences.

### Dog characteristics as risk factors

Several dog characteristics have been found to be associated with SRBs, but without entirely consistent findings. For example, previous research has suggested that mixed breed dogs are more likely to display SRBs than pure breed dogs (McCrave 1991; Flannigan & Dodman 2001) or that specific breeds, such as Cocker Spaniels (Storengen *et al.* 2014) or Golden Retrievers (van Rooy *et al.* 2018), may be more likely to express specific SRBs than other breeds. However, such variations may be due to methodological differences such as case definitions (owner-reported separation anxiety vs diagnosis of separation anxiety at a veterinary clinic), or differing breed compositions of the sample populations (Sherman & Mills 2008), leading to sampling bias which may have influenced findings.

Similarly, previous work has suggested that male dogs are significantly more likely to exhibit SRBs than female dogs (Bradshaw *et al.* 2002; McGreevy & Masters 2008), but this finding is by no means universal, with some finding no association between the sex of the dog and SRBs (Flannigan & Dodman 2001; Mendl *et al.* 2010b; Palestrini *et al.* 2010). Neutered dogs have been found to be more likely to show SRBs than intact dogs (Flannigan & Dodman 2001; McGreevy & Masters 2008). Neutering might affect males and females differently, however, as a questionnaire-based study reported that neutered male dogs were more likely to show SRBs than intact male dogs, while neutered female dogs had lower SRB scores than intact female dogs (van Rooy *et al.* 2018). While the effect of neutering on SRB occurrence requires further clarification, the direction of causation should be considered; owners of dogs with SRBs may be more likely to have their dogs neutered (van Rooy *et al.* 2018), as some veterinarians may suggest neutering male dogs to control problematic or undesirable behaviours (Salman *et al.* 2000).

### Owner characteristics and behaviour as risk factors

Owner experience may be related to the occurrence of SRBs. Dogs belonging to first-time owners were, perhaps surprisingly, less likely to show separation-related defaecation than those belonging to more experienced owners (Jagoe & Serpell 1996). In terms of owner attitudes, owners who were self-reportedly ‘attachment avoidant’ (owners who were classed as dismissive or less sensitive to their dogs’ needs according to a human attachment questionnaire; Collins 1996; Konok *et al.* 2015) had dogs with increased SRBs, possibly because these owners may be less responsive or less consistent in their interactions with their dogs (Konok *et al.* 2015).

Owner approach to training and discipline may additionally be associated with likelihood of SRBs. Less separation-related toileting has been reported with increasing formality of owner-reported obedience training (absent, informal, or formal) (Jagoe & Serpell 1996). However, results are mixed, with González-Martínez *et al.* (2019) finding no association between puppy class attendance and SRBs (González-Martínez *et al.* 2019). Training techniques are generally categorised as either positive or negative, and as either reinforcement- or aversive/punishment-based (Blackwell *et al.* 2012). Many owners tend to use a mixture of two or more of these methods at a time leading to some referring to training techniques as being reward-based (positive reinforcement and negative punishment) or aversive-based (positive punishment or negative reinforcement) (Blackwell *et al.* 2012). Aversive training methods have been associated with increased fear and anxiety (Blackwell *et al.* 2008; Arhant *et al.* 2010), and more owner-reported behavioural problems (Mills 1997; Hiby *et al.* 2004; Arhant *et al.* 2010; Casey *et al.* 2014; Bräm Dubé *et al.* 2020). In terms of SRBs, one C-BARQ (Canine Behavioral Assessment and Research Questionnaire) study found that dogs had worse SRB scores if their owners engaged in more aversive training (Hsu & Serpell 2003). However, a smaller questionnaire-based study found no association between SRB occurrence and training methods (Blackwell *et al.* 2008), indicating a need for clarification, especially if owner training approach can help to prevent this welfare problem. Much of the advice for treating SRBs includes not punishing undesired behaviour upon owner return (Blackwell *et al.* 2006), but its inclusion in advice packages has been questioned, as the advice was not found to contribute to the reduction of pre-existing SRBs in an experimental study of eight dogs (Butler *et al.* 2011). Whilst there are differences between studies in the efficacy of stopping punishment for treating SRBs, it is widely considered to be important to avoid exacerbating anxiety through fear of owner return as well as departure, and is also part of wider evidence that punishment-based techniques negatively impact upon dog welfare and are ethically unacceptable (Blackwell *et al.* 2008; Arhant *et al.* 2010).

In addition to advice on punishment, owners are often advised to avoid excessive greeting behaviours upon owner-dog reunion (Blackwell *et al.* 2006, 2016). There is support for this advice as McGreevy and Masters (2008) found that dogs belonging to owners who played with them within the first 30 min of returning had higher odds of displaying SRBs, suggesting owner interactions upon owner return influence SRBs (McGreevy & Masters 2008). However, the direction of causation is unclear; it is possible that owners of dogs with SRBs perceive their dogs’ SRBs and respond by greeting them effusively or playing with them to comfort them.

Previous research has pointed to the general influence on dog welfare of factors such as sleeping routines, crates and training (Jagoe & Serpell 1996; Podberscek *et al.* 1999; Sherman & Mills 2008). Research has suggested that allowing dogs to sleep in a bedroom or on the owner’s bed could potentially exacerbate behavioural problems (Podberscek *et al.* 1999). Similarly, Jagoe and Serpell’s (1996) questionnaire-based study found that separation-related toileting was more likely to occur when dogs slept in the same room as their owner. Also, a descriptive study found that just over half of a population of dogs diagnosed with separation anxiety at a behaviour clinic slept with their owners, although no comparison with dogs without SRBs was made (Storengen *et al.* 2014). In contrast, one previous study reported no association between SRBs and allowing dogs to sleep on the owner’s bed (Flannigan & Dodman 2001). Learning more about whether, and in what context, puppies come across such experiences during the first few months in new homes, may illuminate more about their influence of later SRB occurrence. Similarly, while owner training approaches and attitudes may influence behaviour, information regarding owner approaches to their dogs at times other than training may shed more light upon how owner interactions influence dog behaviour long-term.

Finally, the home environment of dogs may affect SRB outcome, with dogs belonging to single adults being more likely to show ‘separation anxiety’ (defined in the paper as when dogs ‘become upset when separated from their owner’) than those in multi-person households (Flannigan & Dodman 2001). Recent research from Brand and colleagues (2024), on a cohort population of dogs purchased during the pandemic, found that dogs who had had another dog in the house when they were a puppy were less likely to have SRBs at 21 months of age than those without another dog in the house as a puppy. This suggests that growing up with another dog in the house may help prevent puppies from developing SRBs (Brand *et al.* 2024); however, this was a novel finding, and the protective effect may vary considerably depending on the relationship between the dogs in question. Similarly, changes in the household have been associated with separation anxiety (Flannigan & Dodman 2001), with increases in household members and owner job changes resulting in a greater likelihood of higher SRB scores (McGreevy & Masters 2008).

### Early life experiences and environment as risk factors

Early life experiences can have long-term effects on behaviour and temperament (Appleby *et al.* 2002). Early experiences are thought to affect the ability of dogs to cope with stress (Foyer *et al.* 2013), and that early traumatic experiences of separation or unpleasant experiences can affect later behaviour (Appleby & Pluijmakers 2003). Dogs have a sensitive period of behavioural development and emotional responses. If puppies do not receive appropriate or adequate socialisation during this time, it can result in future behavioural problems. This time-period, known as the ‘socialisation period’, occurs when puppies are 3–12 weeks of age (Scott & Fuller 1965). Tiira and Lohi (2015) found that dogs reported by their owners to be ‘fearful’ had had poorer maternal care and fewer socialisation experiences when they were puppies (Tiira & Lohi 2015), but they did not assess SRBs specifically.

Perhaps relatedly, the provenance of puppies can influence their behaviour long-term. For example, evidence from questionnaire responses have indicated that dogs from pet shops not only had overall worse behaviour and were more aggressive and fearful, but also showed more SRBs than dogs from non-commercial breeders (McMillan *et al.* 2013; Pirrone *et al.* 2016). Similarly, high levels of SRBs were reported in dogs acquired from shelters or rescue centres (Flannigan & Dodman 2001; Blackwell *et al.* 2008; van Rooy *et al.* 2018) and pet shops (McMillan *et al.* 2013; van Rooy *et al.* 2018), while dogs acquired from friends and family may have lower risks of developing SRBs (McGreevy & Masters 2008).

There appears to relatively little research into the potential effect of early life experiences on later SRBs specifically. As mentioned previously, early research identified that puppies become distressed when separated from their mothers or littermates, or when placed in a strange environment, and that puppies were more distressed if they had not experienced being separated before 9–12 weeks of age (Elliot & Scott 1961). This implies that early experiences of being briefly left alone may be helpful to prevent SRBs later on, and puppies may develop issues with being left alone if they have not been habituated to short periods of isolation from a young age (Appleby *et al.* 2002). The influence of breeder environment was investigated in a study which found that dogs obtained from breeders categorised as ‘less responsible’ by the authors due to their rearing practices, had higher levels of behavioural problems, including SRBs (Gray *et al.* 2016), although the exact definition of what constituted ‘responsible’ was not clear.

Programmes to increase socialisation at an early age seem to be effective at reducing SRBs. For example, Vaterlaws-Whiteside and Hartmann (2017) implemented a socialisation programme with litters of puppies at an assistance dog breeding centre. Results indicated that with increased dog-handler, one-on-one time over the first six weeks of life puppies showed better SRB scores (i.e. less evidence of SRB) derived from questionnaires administered to dog handlers after eight months (Vaterlaws-Whiteside & Hartmann 2017). However, in another questionnaire study, whilst general fearfulness was increased in dogs who had sub-optimal early experiences (e.g. poor maternal care and socialisation), lower daily exercise was the only factor that significantly increased SRBs specifically (Tiira & Lohi 2015).

### Aims of the study

We aimed to explore early life risk factors for the development of SRBs, with a view towards making recommendations for future research and prevention strategies. Using a longitudinal questionnaire study of pet dogs living in the UK and Republic of Ireland (ROI), we carried out an exploratory risk factor analysis to investigate and quantify associations between dog and owner factors and specific environmental and dog experiences in early life (up to six months of age) and their effect on owner-reported SRB occurrence at six months of age.

## Materials and methods

### Subjects

Data used in this analysis were collected as part of ‘Generation Pup’, an ongoing large, longitudinal questionnaire-based project, primarily investigating risk factors for dog health and behaviour. Owners living in the UK or the ROI, aged 16 years or older, with puppies of any breed or cross breed are asked to sign up to the study providing that their puppy is aged under 16 weeks at the time of recruitment, or up to 21 weeks of age at registration if entering the UK or ROI through quarantine. Further details on the methodology related to the project (e.g. time-points for surveys, length of time surveys were available for) can be found elsewhere (Murray *et al.* 2021). Owners answer questionnaires on their dogs’ health and behaviour throughout their dogs’ lives. Owners can elect to join the Generation Pup project, and awareness of the project is promoted in a number of ways, including through printed flyers at veterinary practices, electronic advertisements on social media, press articles, dog training classes (Murray *et al.* 2021) and stalls at public events such as Discover Dogs. If an owner had more than one puppy enrolled in the study, one puppy was selected at random to avoid clustering at the level of the household.

### Ethical approval and considerations

All data used for risk factor analyses were obtained from the ‘Generation Pup’ database. Puppies included in the study were born between March 2017 and November 2018. The ‘Generation Pup’ project received ethical approval from the Social Science Ethical Review Board at the Royal Veterinary College (URN SR2017-1116), the University of Bristol Animal Welfare Ethical Research Board (UIN/18/052) and the Dogs Trust Ethical Review Board (ERB009). All animals included in the study were dogs belonging to owners who voluntarily signed up to ‘Generation Pup’ to answer questionnaires regarding their dog’s health and behaviour. ‘Generation Pup’ owners are directed to contact a clinical animal behaviourist via specific websites (www.asab.org.uk and www.apbc.org.uk) if they are concerned about their dogs’ behaviour but are advised that ‘Generation Pup’ researchers cannot provide direct help or advice regarding their dogs’ behaviour.

### Data collection

Data for the current study were comprised from selected owner responses to ‘Generation Pup’ questionnaires completed upon signing up to the study (‘About Me’, ‘About My Puppy’, and ‘About My Household’), at ‘Settling In’, 12 weeks, 16 weeks, and six months (Table 1) (questionnaire files are available as supplementary material in Murray *et al.* 2021). Owners answered questions regarding the early life of their puppies (≤ 16 weeks) by filling out one to three questionnaires depending on puppy age at registration (Table 1). The timings of the questionnaires used in the present study are shown in Table 1 (for further details, see Murray *et al.* 2021).

**Table 1.** List of ‘Generation Pup’ questionnaires used for the present study and the corresponding age that dogs reached when owners received each questionnaire

‘Generation Pup’ questionnaires	Puppy age at time of questionnaire completion
‘About Me’ and ‘About My Household’	Before puppy registration to study
‘About My Puppy’	At point of registration onto the study
‘Settling In’ (SI)*	1–3 weeks after acquisition, or until 12 weeks of age, whichever was sooner
12-week*	12 weeks (84 days) to 15.5 weeks (108 days)
16-week*	16 weeks (112 days) to 19.5 weeks (136 days)
SI/12-week combined*	Between age 12 weeks (84 days) and 15.5 weeks (108 days).
SI/12-week/16-week combined*	Between 15.5 weeks (109 days) and 16 weeks (112 days)
Six-month	6 months (180 days) to 6 months + 3.5 weeks (204 days)

* Questionnaires from which ‘≤ 16 weeks’ time-point data were extracted. Data from the latest time-point available for each question were used.

Depending on when owners acquired their puppies, owners completed differing combinations of the ‘Settling In’, 12-week and 16-week questionnaires, each of which contained similar, overlapping questions (Table 1; Murray *et al.* 2021). This meant that equivalent questions across the three questionnaires needed to be condensed down to create only one response per dog. Therefore, for each dog, only the latest available response from the three questionnaires was taken. This created a new ‘≤ 16 weeks’ time-point for some variables.

### Collection of risk factor data

Information about early life factors and owner-dog interactions was gained from questions across the ‘About Me’, ‘About My Puppy’, ‘About My Household’, ≤ 16 weeks (see above/Table 1 footnote), and six-month ‘Generation Pup’ questionnaires (Table 1). Most questions, but not all, were compulsory (throughout all time-points). Questions were presented with multiple choice or ‘tick one box’ options and/or as free-text options and were either completed online or on paper copies. Each survey included questions supporting more than one study, so the overall aim of the ‘Generation Pup’ questionnaires of exploring potential risk factors for dog health and behavioural problems throughout dogs’ lives (Murray *et al.* 2021) was communicated to respondents, rather than the specific aim of the current study. This may have minimised response bias towards answers that owners believed might align with preventing SRBs. However, because each survey took an estimated 20 to 30 min to complete (Murray *et al.* 2021), to motivate respondents to complete later sections about SRBs, respondents were told that their answers would ‘help us make recommendations that can be used to help dogs adjust to being left alone in the future’. Certain questions required the owner to report how they had responded to behaviour in the last seven days only, to reduce recall bias. Responses regarding puppy factors (e.g. source, breed, sex, and age joined household), owner factors (age, education, experience with dogs), experiential factors (sleep, house-training, experiences) and owner responses to training and behaviour across the early questionnaires, at ≤ 16 weeks and at six months were considered as potential risk factors (see Table S1; Supplementary material).

### Six-month questionnaire: SRB case definition

The six-month ‘Generation Pup’ questionnaire was used to identify which dogs did, or did not, perform SRBs at six months of age, based on owner-report of dog behaviour. It included a section entitled ‘My puppy’s day’ which comprised questions on dog responses to several common situations including when the puppy was left alone without human company (Murray *et al.* 2021). Relevant questions which were used to define the dependent variable for analysis included those on how dogs behaved (Table 2): when the owner was relaxing with their dog; when the dog was left alone without human company; when the owner was not paying attention to their dog; and when the owner was separated from the dog by a baby gate or door. Dog behaviour when left alone was split into two questions: how the dog behaved when left alone without human company; and how the dog behaved upon owner return following a period of separation (Table 2, Scenario 2 question 1 & 2). Owners were presented with a list of possible behaviour responses to these scenarios and, for each behaviour, owners were asked to tick one of four options: ‘Definitely yes’, ‘Definitely no’, ‘I don’t know’, or ‘Not applicable’. For each question ‘Other (please specify)’ was included as a possible response where owners could fill in a free-text response. The wording of the relevant questions used to create the case definition, and the presented scenarios are listed in Table 2.

**Table 2.** Scenarios, question wording and potential responses from the ‘Generation Pup’ six-month questionnaire used to create the case definition for SRB occurrence in puppies at six months of age. Three scenarios were presented to the owners, as follows. Scenario 1: Owner relaxing with dog; Scenario 2: Dog left alone without human company (split over two questions); and Scenario 3: Owner not paying attention to dog.

Scenario 1	Question wording
Owner relaxing with dog	During the last seven days, when I/we were relaxing at home with my puppy, he/she often (at least daily)…
**Behaviour response options:**	
Rolled on his/her back Spun or turned in a circle Paced/ran back and forth Barked Growled Howled Trembled or shook Pined, whined, or cried Hid/cowered Stayed close/came to/tried to get attention from me or other household member Followed me around the house Leant on me Avoided being left on his/her own in a room Chewed his/her own toys	Scratched/damaged around the door or entrance in to and out of the house Scratched/damaged furniture Scratched/damaged skirting boards Chewed/damaged other things which belong to him/her (for example his/her bed) Chewed/damaged items that belong to people in the household Chewed or intensively licked parts of his/her own body (for example tail, flank, or paws) Weed inside the house or where he/she shouldn’t Pooed inside the house or where he/she shouldn’t Dribbled/drooled Began panting (when not particularly hot) Jumped up at me Other (please specify)

Scenario 2	Question wording
Dog left alone without human company (question 1)	In general, during the last seven days, when my puppy had been left at home WITHOUT human company, I/we returned to the house to find that he/she had…
**Behaviour response options:**	
Eaten a treat Chewed his/her own toys Scratched/damaged around the door or entrance in to and out of the house Scratched/damaged furniture Scratched/damaged skirting boards Chewed/damaged other things which belong to him/her (for example his/her bed) Chewed/damaged items that belong to people in the household	Chewed or intensively licked parts of his/her own body (for example tail, flank, or paws) Been in his/her bed Weed inside the house or where he/she shouldn’t Pooed inside the house or where he/she shouldn’t Vomited (when otherwise not ill) Dribbled/drooled Other (please specify)

Scenario 2	Question wording
Dog left alone without human company (question 2)	In general, during the last seven days, when my puppy had been left at home WITHOUT human company and I/we returned to the house, he/she…
**Behaviour response options:**	
Greeted me/us Had a low/straight (relaxed) tail posture Had a raised tail posture Had a wagging tail Held tail down between his/her legs Was interested but stayed calm Was disinterested/didn’t react/rested or slept Had been watching/waiting at the window Rolled on his/her back Spun or turned in a circle If available, chose to go to his/her bed or enclosed space (for example an indoor crate) Paced/ran back and forth Barked Growled/snarled/raised upper lip	Howled Froze or stayed still Trembled or shook Pined, whined, or cried Hid/cowered Ran/pulled away Followed me around the house Stayed close/came to/tried to get attention from me or other household member Jumped up at me Bit or snapped at me Picked up his/her own toys Began panting (when not particularly hot) Other (please specify)

Scenario 3	Question wording
Owner not paying attention to dog	In general, during the last seven days, when I or other members of the household were not paying any attention to my puppy, he/she…
**Behaviour response options:**	
Was disinterested/didn’t react/rested or slept Spun or turned in a circle Lay or stood or sat in front of the door If available, chose to go to his/her bed or enclosed space (for example an indoor crate) Paced/ran back and forth Barked Growled/snarled/raised upper lip Howled Pined, whined, or cried Stayed close/came to/tried to get attention from me or other household member Followed me/us around the house Chewed his/her own toys Scratched/damaged around the door or entrance in to and out of the house Scratched/damaged furniture Scratched/damaged skirting boards	Chewed/damaged other things which belong to him/her (for example his/her bed) Chewed/damaged items that belong to people in the household Chewed or intensively licked parts of his/her own body (for example tail, flank, or paws) Weed inside the house or where he/she shouldn’t Pooed inside the house or where he/she shouldn’t Began panting (when not particularly hot) Jumped up at me Bit or snapped at me Brought toys over to play Had a ‘mad five minutes’ Tried to sit with me on a chair or sofa Tried to get on my lap Picked up items from the floor Grabbed at my clothes (for example sleeves) Other (please specify)

SRB: Separation-related behaviours.

### Missing data

Some dogs had missing values for different variables meaning the sample size available varied for each explanatory variable. ‘Prefer not to answer’ responses were treated as missing data as appropriate for relevant questions.

### Data processing

#### Data recategorisation for analysis

Variables were combined as necessary to allow comparisons between categories. Adjacent or similar categories were combined where possible if one or both contained low numbers of respondents (categories with less than ten individuals) and if combining categories made logical sense. For example, at univariable analysis only five individual breeds were compared to unknown crossbreeds, known crossbreeds and other pure breeds due to low sample size in other breed categories. Further condensed categories were then created depending on results of initial univariable analyses to allow variables to be investigated further. For example, breed data were further condensed into crossbreeds, Labrador Retrievers, and other pure breeds as there was no evidence of significant associations between other pure breeds and SRBs. The new categories derived from the original questionnaire data are described in Tables S1 and S2 in the Supplementary material.

Owners could choose from a series of potential responses for correcting behaviour they would like to discourage in a number of different scenarios (when the owner was not giving the dog attention, when separated by a baby gate, relaxing at home with the owner, and when the owner returned to the household) ranging from telling the dog off to cuddling them (Table 3), in any combination. Comparison of the four different types of training (positive punishment, negative reinforcement, positive reinforcement, negative punishment) (Blackwell *et al.* 2012) was thus challenging (categorisation summarised in Table 3). Therefore, for simplicity, owners who indicated they used two or more positive punishment techniques (referred to here as aversive techniques) were compared to owners who used fewer than two positive punishment techniques. Owners were categorised this way to try and prevent including owners who had, for example, shouted at their dog once, with owners who generally used aversive techniques when interacting with their dog.

**Table 3.** Categorisation of owner responses to questions regarding correcting their puppy for ‘bad’ behaviours in different scenarios (when owner not giving the dog attention, separated by a baby gate or door, relaxing at home with the owner, when owner returned to the household) from the ‘Generation Pup’ six-month questionnaire according to training categories as described in Blackwell *et al.* (2012)

Training technique	Definition	Owner responses to ‘bad’ behaviour	Classification in analysis
Positive punishment	Likelihood of a behaviour decreased by associating the behaviour with an aversive stimulus	Tell him/her off Use a physical reprimand/correction (for example smack or tap him/her on nose or bottom) Distract him/her (for example with a rattled can/loud noise/water spray)	Aversive
Negative reinforcement	Likelihood of a behaviour increased by removing an aversive stimulus	Physically manipulate him/her (for example hold still, put into a sit, or push off if he/she jumps up) Hold him/her still	Aversive
Positive reinforcement	Likelihood of a behaviour increased when by associating the behaviour with a rewarding stimulus	Play with him/her Stroke him/her Give him/her a food treat Cuddle him/her Encourage him/her to calm down	Non-aversive
Negative punishment	Likelihood of a behaviour decreased by associating behaviour with removal of rewarding stimulus	Move away or withhold affection Don’t give him/her treats that would normally be given at this time Ignore him/her Move him/her to another part of the house	Aversive (‘time-out’)

Categories within variables were also recategorised to prevent multicollinearity as identified by inflated standard errors during initial univariable analysis. For example, at ≤ 16 weeks owners were asked what techniques they used to reward their puppy during training (Table S1; Supplementary material). Owners were able to tick any of the following in any combination: verbal praise, physical praise, a toy, a ball, a clicker, some of his/her normal dog food, a novel dog food (‘a dog food other than his/her own kibble/biscuit’), dog food treats, human food, none/no other rewards are used, training has not started or other. For the purposes of analyses, these responses were categorised into owners who ticked dog treats or novel kibble, or owners who did not tick either of these options following initial univariable analysis of the binary reward categories. This was carried out to try and capture differences in approaches to reward techniques by combining those categories which had reached threshold significance in initial univariable analysis screening. Miscellaneous responses were categorised into one of the existing categories where possible; otherwise, responses were coded as ‘other’.

### Case definition

Vocal, destructive (Bradshaw *et al.* 2002), repetitive behaviours (Blackwell *et al.* 2006) and inappropriate toileting (McCrave 1991) are commonly reported canine SRBs, so these behaviour categories were used to define cases. To create a binary case/control outcome variable for logistic regression analysis, ‘Generation Pup’ six-month questionnaire responses were used to categorise dogs as ‘cases’ if they were reported to show one or more of the following behaviours when left alone without human company:

• Spun or turned in a circle;
• Paced/ran back and forth;
• Barked;
• Howled;
• Pined, whined, or cried;
• Scratched/damaged around the door or entrance in to and out of the house;
• Scratched/damaged furniture;
• Scratched/damaged skirting boards;
• Chewed/damaged other things which belong to him/her (for example his/her bed);
• Chewed/damaged items that belong to people in the household;
• Weed inside the house or where he/she should not;
• Pooed inside the house or where he/she should not.

Dogs displaying these behaviours when people were in the household, e.g. not giving the dog attention, separated by a baby gate, relaxing at home, were excluded as cases. Cases were thus classed as dogs who were reported to display any one or more of the SRBs listed above, but only when the dog was left alone, but not when the owners remained within the home.

Free-text responses were recoded and allocated to pre-existing categories where appropriate, and missing data and ambiguous cases/controls were excluded (e.g. when an owner provided information regarding their puppy’s behaviour when left alone in the car, rather than when left alone in the home).

### Control definition

Control dogs were those who did not show any of the 12 behaviours listed above when left alone (indicative of SRB) or separated at home by a door or baby gate (an ambiguous situation where dogs may be separated from owners and may or may not be able to see/be aware of the owners’ presence in the household), but they could display any of these behaviours in the other two contexts: when the owner was relaxing or not paying attention to the dog.

### Statistical analysis

#### Univariable logistic regression analysis

The SRB case definition (case; control) was used as the dependent variable in analyses. Univariable logistic regression analysis was conducted for each potential risk factor from the ‘About My Puppy’, ‘About My Household’, ‘About Me’, ‘Settling In’, ≤ 16-week, and six-month ‘Generation Pup’ questionnaires. Non-modifiable factors, including dog sex, dog breed, and owner age were also investigated as potential risk factors. Explanatory variables which were liberally associated with SRBs (*P* ≤ 0.200) were taken forwards for multivariable analysis (Table S2; Supplementary material).

It should be noted that, while research has suggested particularly high levels of reported SRBs in dogs purchased during the COVID-19 pandemic (often known as ‘Pandemic Puppies’) (Brand *et al.* 2024), potentially due to reduced time left alone during national lockdowns (Harvey *et al.* 2022), data collection for this study was conducted prior to this time-period and therefore this effect was not investigated during analyses.

#### Multivariable logistic regression model selection

A multivariable logistic regression model, with the SRB response variable, was built using a forward stepwise model selection process. Variables were added to the model in order of *P*-value obtained in univariable analyses in ascending order. After each variable of interest was added to the model, an online Chi-squared calculator (Social Science Statistics 2020) was used to calculate whether the difference in Chi-squared values and degrees of freedom suggested the model was significantly improved by the addition of that variable; if significant, the variable would be retained in the model. Variables were added to the model until all variables were statistically significant (*P* < 0.050) and/or it was determined that the model fit was improved with the inclusion of a particular variable according to the Chi-squared calculator (Social Science Statistics 2020).

All analyses were conducted in the IBM SPSS® Statistics package (Version 26).

## Results

### Description of the sample

There were data for dogs belonging to a total of 1,987 respondents included in the original six-month dataset downloaded from ‘Generation Pup’. Dogs were excluded if they not been left alone without human company at six months (162 dogs), if key data were missing (1,218 dogs), or if data were ambiguous across response options for the case definition (e.g. ‘I don’t know’ or ‘Not applicable’ across all answer options) and if they did not fit the case definitions for either the cases or controls (462 dogs). Of the resulting 145 dogs included in the final model, there were 108 pure breed dogs (74.5%) and 37 cross breed dogs (25.5%). The most common breed was the Labrador Retriever, and the most common Kennel Club group for purebreds was gundog. There were slightly more male (52.4%) than female dogs. The mean (± SD) age at acquisition, excluding homebred puppies, was 61.9 (± 12.5) days; median = 58 days, equating to approximately eight weeks and six days old. Demographics of both dogs and owners are shown in Table 4.

**Table 4.** Demographics of dogs (n = 145) and owners included in the final multivariable model investigating risk factors for canine SRBs at six months of age, using owner-reported data from the longitudinal study ‘Generation Pup’. Categories without a natural scale are ordered from most to least common, followed by miscellaneous categories

Population	Variable	Category	Description n (%)
Dog	Sex	Male	76 (52.4)
		Female	69 (47.6)
	Source	A kennel club assured breeder	43 (29.7)
		A professional breeder	23 (15.9)
		A hobby or occasional breeder	58 (40.0)
		A charity/rescue/adoption/rehoming organisation	5 (3.4)
		Homebred	5 (3.4)
		Other	11 (7.6)
	Breed	Labrador Retriever	20 (13.8)
		Border Collie	10 (6.9)
		Cocker Spaniel	9 (6.2)
		English Springer Spaniel	6 (4.1)
		Golden Retriever	5 (3.5)
		Other pure breeds comprising <5 dogs	58 (40.0)
		Crosses of known breeds	28 (19.3)
		Unknown cross breed	9 (6.2)
	Kennel club groupings	Gundog	47 (32.4)
		Pastoral	16 (11.0)
		Terrier	13 (9.0)
		Toy	10 (6.9)
		Working	7 (4.8)
		Hound	6 (4.1)
		Utility	5 (3.5)
		None	41 (28.3)
	Age at acquisition	8+ weeks of age	108 (74.5)
		<8 weeks of age	32 (22.1)
		Homebred	5 (3.5)
Owner	Gender	Female	134 (92.4)
		Male	11 (7.6)
	Age category (years)	16–24	6 (4.1)
		25–34	16 (11.0)
		35–44	40 (27.6)
		45–54	30 (20.7)
		55–64	46 (31.7)
		65+	7 (4.8)
	Marital status	Married	97 (66.9)
		Single	21 (14.5)
		Cohabiting /Common Law-Partnered	14 (9.7)
		Divorced/Separated	8 (5.5)
		Widowed	2 (1.4)
		Preferred not to say	2 (1.4)
		Civil Partnered	1 (0.7)
	Experience owning dogs	Had always owned a dog, both as a child and adult	39 (26.9)
		Almost always had a dog, as a child and adult, but with some breaks	35 (24.1)
		Had previous dogs as an adult only	32 (22.1)
		First time ever	17 (11.7)
		First time as an adult, but had a family dog as a child	15 (10.3)
		Other^a^	7 (4.8)

SRB: Separation-related behaviours.

a Owner ticked the ‘Other’ option from the list of answers and provided further information. Examples of answers included information not relevant to the question, for example ‘Currently also have an adult dog’, or ‘Currently have 6 dogs’.

Almost one-third of owners were aged between 55 and 64 years, with the next most common age group being 35–44 years (Table 4). Most owners (73.1%) reported that they had owned at least one previous dog as an adult, whilst 22.0% reported that this was the first dog they had owned as an adult.

### Description of separation experiences

**Figure 1.**
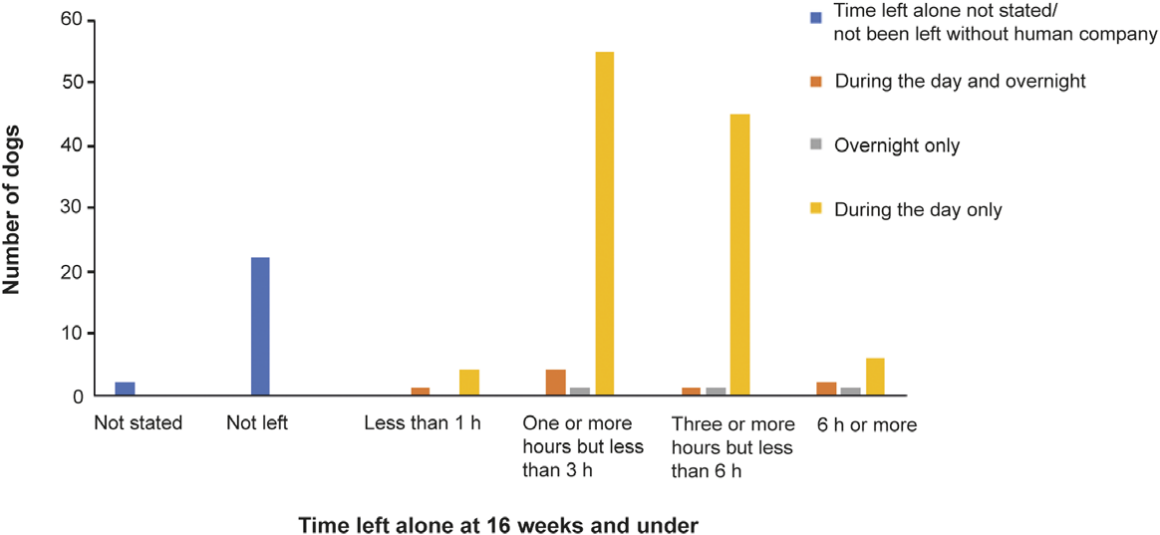
The longest continuous duration for which dogs (n = 145) were left alone at 16 weeks and under in a study investigating risk factors for canine SRBs at six months of age, using owner-reported data from the longitudinal study, ‘Generation Pup’.

At ≤ 16 weeks, most dogs (110; 75.9%) had been left alone during the day, eight (5.5%) had been left during both the day and overnight, and three dogs (2.1%) had been left alone overnight only. In contrast, 22 dogs (15.2%) had not been left alone without human company at all. Most dogs had been left for a maximum of 1–6 h (Figure 1).

**Figure 2.**
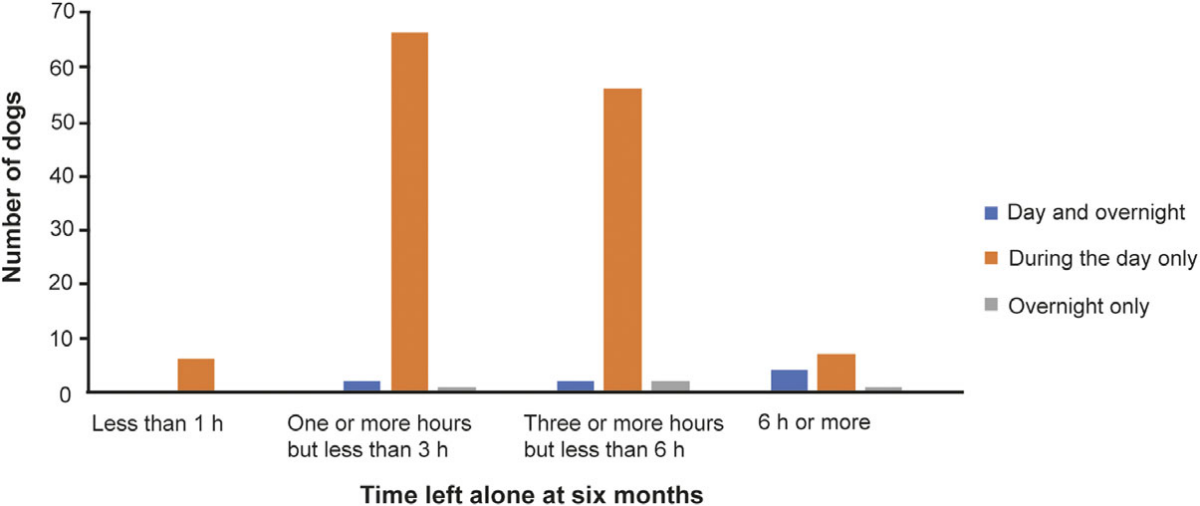
The longest continuous duration for which dogs (n = 145) were left alone in a study investigating risk factors for canine SRBs at six months of age, using owner-reported data from the longitudinal study, ‘Generation Pup’. Dogs were left during the day only (orange), overnight only (grey) and during the day and overnight (blue).

At the six-month time-point, all dogs had experienced being alone: 133 dogs (91.7%) had been left without human company during the day only, eight (5.5%) had been left alone both during the day and overnight, and four (2.8%) had been left alone overnight only. As at the ≤ 16-week time point, most dogs were left alone for a maximum of 1–6 h (Figure 2).

Sixty-eight of the puppies (46.9%) displayed SRBs at six months of age according to the case definition. Table 5 shows the number of dogs performing each SRB, only when left alone. The most commonly reported SRB was pacing (14.5%), followed by whining (7.6%) and spinning/circling (6.9%).

**Table 5.** Number of dogs (n = 145) performing each canine SRB of interest at six months of age (only when left alone) using owner-reported data from the longitudinal study Generation Pup. The SRBs are arranged from most to least commonly reported.

SRB included in case definition	Dogs displaying SRB only when left alone (n [%])
Paced/ran back and forth	21 (14.5)
Pined, whined, or cried	11 (7.6)
Spun or turned in a circle	10 (6.9)
Chewed/damaged other things which belong to him/her (e.g. his/her bed)	7 (4.8)
Scratched/damaged furniture	6 (4.1)
Scratched/damaged around the door or entrance in to and out of the house	6 (4.1)
Weed inside the house or where he/she shouldn’t	6 (4.1)
Scratched/damaged skirting boards	5 (3.5)
Chewed/damaged items that belong to people in the household	4 (2.8)
Pooed inside the house or where he/she shouldn’t	3 (2.1)
Howled	2 (1.4)
Barked	1 (0.7)

SRB: Separation-related behaviours.

### Multivariable analysis

The final model consisted of eight explanatory variables, explaining 0.526–0.702 of the total variation (Cox & Snell R Square to Nagelkerke R Square values). The final model is shown in Table 6 (univariable results are available in Table S2; Supplementary material).

**Table 6.** Multivariable logistic regression model for risk factors for canine SRBs at six months of age, using owner-reported data from the longitudinal study ‘Generation Pup’ (n = 145). Significance threshold: *P* < 0.050

Variable Name	Reference Category	Comparison Categories	Odds Ratio	95% CI	P-value
Use of enclosed Space at ≤16 weeks	Not enclosed at ≤16 weeks	(Reference)	1.00		<0.001
		Daytime only (overnight not mentioned)	0.245	0.03–1.78	0.164
		Overnight experience of being enclosed	0.029	0.00–0.24	<0.001
Owner Age Category	35 years and older	16 to 34 years old	11.12	1.51–81.67	0.018
Training Reward Used at ≤16 weeks	No kibble other than normal kibble and/or dog food treats	Kibble other than normal kibble and/or dogs food treats	13.85	2.81–68.33	0.001
House Training Progression at ≤16 weeks	Zero to three accidents a week	Is ongoing, but there are still four or more accidents per week	13.25	2.49–70.37	0.002
Response to Bad Behaviour when puppy receiving No Attention at Home at six months	Bad behaviour shown and ≤1 aversive techniques^a^	(Reference)	1.00		0.029
		Bad behaviour shown and ≥2 aversive techniques^a^	11.09	1.72–71.54	0.011
		Bad behaviour not shown	5.35	0.89–32.04	0.066
Maximum Hours of Sleep at Night at ≤16 weeks	6–8 hours	(Reference)	1.00		0.002
		9 hours	0.142	0.03–0.72	0.018
		10+ hours	0.019	0.00–0.16	<0.001
		Unspecified	0.39	0.09–1.62	0.192
Response to Bad Behaviour when Relaxing at Home at six months	≤1 aversive techniques^a^	(Reference)	1.00		0.001
		≤1 aversive techniques^a^ but use time-out methods^b^	97.96	8.36–1148.54	<0.001
		≥2 aversive techniques^a^	27.96	3.01–259.96	0.003
Response to Bad Behaviour upon Return (six months)	Other responses	(Reference)	1.00		0.003
		Fuss (Greet/Stroke/Cuddle/Play)	5.76	1.24–26.71	0.025
		Bad behaviour not shown	0.33	0.08–1.32	0.118

SRB: Separation-related behaviours.

a Aversive techniques: Tell him/her off, physically reprimand/correct him/her (for example smack or tap on nose or bottom), distract him/her (for example with a rattled can/loud noise/water spray), physically manipulate him/her (for example hold still, put into a sit, or push off if he/she jumps up), hold him/her still.

b Time-out methods: Move away from him/her, don’t give him/her treats that would normally be given at this time, ignore him/her, move him/her to another part of the house.

Dogs had significantly lower odds of SRBs if they had experienced being in an enclosed space at night at ≤ 16 weeks compared to dogs who had not been in an enclosed space at this time-point. Those who had 9+ h of sleep at night at ≤ 16 weeks had significantly decreased odds of showing SRBs at six months, compared with those who slept a maximum of 6–8 h at this time. Dogs were more likely to show SRBs if they were not yet fully house-trained at ≤ 16 weeks. The odds of SRBs were also increased if owners used special kibble or treats during training at ≤ 16 weeks (Table 6) and were greatly increased if they used two or more aversive training techniques, or time-out methods (negative punishment) at six months across different scenarios (when their puppy was receiving no attention and relaxing, respectively; Table 6). Owners who ‘fussed’ over (greeted/stroked/cuddled/played with) their dogs at six months in response to ‘bad’ behaviour when returning to the household also saw increased odds of their dogs showing SRBs at six months (Table 6).

Additionally, dogs belonging to younger owners (aged 16 to 34 years) had increased odds of SRBs compared to owners aged 35 years and older. No other owner or dog characteristics reached statistical significance in the final model.

## Discussion

This is the first study, to the authors’ knowledge, that has explored the potential effects of early life risk factors, dog-owner interactions, and general dog and owner risk factors, for SRBs at six months of age. The results suggest that overnight conditions in the early lives of puppies and owner interactions with their puppies are important factors influencing SRB development. More specifically, experience of being in an enclosed space overnight and increased sleep at ≤ 16 weeks appear to serve as a protective function against SRB development at six months of age. Conversely, younger owners, poor house-training progression, and aversive approaches to training used at this time may increase the likelihood of SRBs. Positive and negative punishment across different situations at six months of age greatly increased a dog’s likelihood of SRBs, as did fussing over dogs upon reunion.

We identified an SRB prevalence of 46.9%, which is higher than previously reported in some owner-report studies (Bradshaw *et al.* 2002; Dinwoodie *et al.* 2019; Brand *et al.* 2024), but comparable to other estimates (Soares *et al.* 2010; Blackwell *et al.* 2016). Differences in populations and ages of dogs, as well as methodologies and case definitions are likely to explain varying prevalence estimates. For example, this study specifically relates to puppies at six months of age, and excluded ambiguous dogs based on stringent case: control definitions. In a questionnaire study, such as this, we might expect to have underestimated SRB prevalence through owners being unaware of SRBs that generate no audible or physical evidence, but it is notable that the most commonly reported SRBs here included covert repetitive behaviours (pacing and spinning). In the six-month ‘Generation Pup’ questionnaire, one of the questions regarding SRBs (Table 2) referred to behaviours the dogs showed upon owner return rather when the owner had been absent. This means the case definition encapsulates both SRBs and behaviours displayed upon initial owner return in response to owner absence, so perhaps the pacing and spinning occurred once owners returned, rather than when they were absent. It is also possible that owners participating in the ‘Generation Pup’ survey are especially vigilant to their dogs’ behaviour generally and may own cameras to observe their dogs when they are alone, for example.

The results apply to SRBs at six months of age only and relate only to the overt behaviours reported to occur during separation, rather than to the dogs’ underlying welfare states, so caution should be exercised in not over-interpreting the findings. Also, the exploratory nature of the study means that many potential risk factors were tested, so it is possible that some results are Type I (falsely significant) (Bender & Lange 2001) and Type II errors (false negative) due to a small sample size and low statistical power (Mascha & Vetter 2018). Nevertheless, the findings of this study could help inform future research in this area. The potential risk factors identified in the present study fall into broad themes and will be discussed in more detail accordingly: enclosed spaces and sleep, house-training, training approaches, owner responses upon reunion, and other remaining factors.

### Enclosed spaces and sleep

In the present study, puppies with experience of being in an enclosed space overnight at ≤ 16 weeks had decreased odds of developing SRBs, compared with puppies with no experience of being in an enclosed space. While a number of sources suggest that providing dogs with a crate or safe place during separation may help to reduce anxiety around owner departure (e.g. Tuber *et al.* 1999; Houpt *et al.* 2007), others have found that crated dogs show more yawning and lip-licking during separation than non-crated dogs (Palestrini *et al.* 2010) and emphasise the importance of ensuring dogs are not forced into enclosed spaces as punishment for bad behaviour (Schwartz 2003). There is little previous evidence to suggest that experience of crates or enclosed spaces, particularly overnight, may be protective against SRBs. However, there is some evidence suggesting that other sleeping experiences may contribute to SRB expression. For example, studies have suggested that allowing dogs to sleep in a bedroom or on the owner’s bed could potentially exacerbate SRBs (Podberscek *et al.* 1999), such as separation-related urination and defaecation (Jagoe & Serpell 1996). However, Flannigan and Dodman found no association between SRBs and allowing dogs to sleep on the owner’s bed (Flannigan & Dodman 2001). Whilst previous research is slightly ambiguous, our results lend weight towards overnight enclosed spaces being associated with reduced SRB development. The result is correlational, and whether dogs sleep alone or with owners has had associations with other putative diagnoses (e.g. Hoppe *et al.* 2017). Recommendations need to be treated with caution, and treatment plans should always be based on the specific diagnosis and treatment plan for individual dogs.

The direction of causation underlying the association between overnight enclosure use and reduced SRBs requires further investigation. One explanation could be that, even if many owners initially attempt to enclose their dogs overnight, this is only successful for those dogs who are less distressed by separation; owners may quickly give up if their dogs show signs of stress. If this is the case, then recommending owners to place puppies in enclosed spaces, such as crates, overnight would not necessarily be constructive. Palestrini and colleagues (2010), in their video study, found that crated dogs showed increased yawning and lip-licking behaviour than those dogs not confined to a crate, and other studies found no association between owner-reported separation anxiety and whether dogs had been crated (Cannas *et al.* 2010; Herron *et al.* 2014).

Alternatively, overnight use of enclosed spaces could be helpful in reducing SRB risk. Overnight experience of spatial restriction may prepare puppies for being alone for longer periods during the day when they are older. Enclosed spaces also enable a degree of separation, albeit with dogs potentially being able to see/hear their owners nearby, thereby creating a step between being with the owner and being entirely alone when owners are absent from the home. Crating puppies, or offering them an alternative enclosed space, if trained with positive reinforcement from an early age, may help to create a safe ‘den’ for them (Houpt *et al.* 2007). Puppies may learn to relax when partially separated from owners in such enclosed places, and associate them with going to sleep, so they may be less distressed at being fully separated from their owner when this occurs later. Our data did not include information on whether or not owners were nearby while puppies were enclosed, nor whether the breeders had carried out sleep or crate training before the puppies went to their homes, but these factors could be important in determining whether enclosed spaces help to reduce SRBs.

We also found that puppies were less likely to develop SRBs if their owners reported that the puppies slept ≥ 9 h at night at ≤ 16 weeks, compared to those who had only 6–8 h of sleep at this time. Dogs have been reported to sleep between 7.7 and 16 h (for a review, see Campbell & Tobler 1984) and puppies tend to spend the majority of their time sleeping or resting (Frank *et al.* 2007; Cannas *et al.* 2010). However, Kinsman and colleagues (2020) reported that puppies at 16 weeks of age slept on average 7 h, while dogs at 12 months slept an average of 7.3 h (Kinsman *et al.* 2020). These data and those of the present study are based upon owner-report so we cannot be sure of their accuracy and whether they are representative of the true sleeping habits of dogs. The likelihood that any over- /under-estimates of sleep are associated with SRB occurrence is likely to be low; any similar research in future could incorporate actual sleep data to increase the validity and accuracy of results.

Our result aligns with previous research showing that dogs without SRBs tended to sleep and rest more than dogs diagnosed with separation anxiety (Scaglia *et al.* 2013). The direction of causality could be either way, with dogs who happen to be less anxious (and thus arguably less likely to perform SRBs) sleeping better, or alternatively with dogs who are enabled to sleep for longer being more resilient to challenges including separation. In humans and rodents, those with disturbed sleep are less likely to be able to cope with stressful environments (Meerlo *et al.* 2008). Encouraging more sleep and relaxation at early ages, for example, by establishing clear routines of activity and rest for puppies during the day (Dogs Trust 2021), could potentially decrease the likelihood of dogs displaying SRBs as they age; further research could investigate this over a longer time-scale.

### House-training

Puppies whose house-training was still ongoing at ≤ 16 weeks, with ≥ 4 accidents per week, had increased odds of displaying SRBs at six months, compared with puppies who had 0–3 accidents per week at that time-point. House soiling is commonly reported in dogs with separation problems (McCrave 1991) and thought to be a sign of general anxiety. Accordingly, toileting was included in the case definition, which could have meant that this result was inevitable or circular. However, the case definition specified that SRBs had to only occur when the puppy was left alone and not in scenarios when the owner was present. There were very few dogs for whom toileting was their only SRB; just 6/68 cases in the study only urinated and/or defaecated without other SRBs when left alone. Another consideration is that the ‘Generation Pup’ question regarded accidents and house-training in general, and the context of these accidents was not specified. Owners may have included separation-related soiling in their estimation of toileting accidents. Thus, this result may rather represent a correlation such that dogs with separation-related toileting at ≤ 16 weeks were also likely to show SRBs at six months. Inappropriate elimination can also occur for many reasons such as medical problems, excitement, anxiety, fear or marking behaviour (McCrave 1991), which should be considered.

### Training approaches

Owners who used ≥ 2 aversive techniques when trying to discourage unwanted behaviours when relaxing at home or when no one was paying attention to their dog, when their dogs were six months of age, had greatly increased odds of their dogs displaying SRBs compared to those who used ≤ 1 aversive training technique in these scenarios. This is in general agreement with the existing literature regarding the relationship between aversive training styles and problem behaviours overall (Hiby *et al.* 2004; Bräm Dubé *et al.* 2020). However, previous results on whether aversive techniques affect SRBs specifically are mixed. For instance, a study that compared dogs trained using reward-based or aversive techniques during the Ainsworth Strange Situation Test, did not find any difference between the two types of training for SRBs, such as whining or exit-directed behaviours (Vieira de Castro *et al.* 2019). Stopping punishment of SRBs upon owner return, among other interventions, did effectively reduce SRB development in newly adopted rescue dogs (Blackwell *et al.* 2016), but some authors have suggested that stopping punishment may not play a large role in reducing SRBs (Butler *et al.* 2011; Herron *et al.* 2014). Intervention programmes also often introduce multiple interventions at once, making interpretation of the efficacy of individual elements challenging (Blackwell *et al.* 2006).

We found that the use of aversive techniques and punishment upon owner return (as opposed to when relaxing or withholding attention) was not significantly associated with SRBs in the final model, despite reaching threshold significance in univariable analyses. While this result could be further investigated with a different dataset, this may suggest that punishment after separation does not necessarily affect SRBs’ outcomes, as suggested by Butler *et al*. (2011). Perhaps owner interactions at other times, such as relaxing and no attention, affect SRB presentation via more general effects on dog anxiety levels. In addition, logically, punishment on return would most likely lead to anxiety about owner return rather than influencing anxiety about owner absence.

We also found that puppies of owners who used ‘time-outs’ when correcting their puppy when the owner was relaxing, also showed increased odds of developing SRBs. Our definition of ‘time-outs’ included negative punishment methods where owner affection or attention was removed (Arhant *et al.* 2010; Blackwell *et al.* 2012). These techniques are sometimes favoured by owners because they perceive them as kinder or less likely to cause fear, than punishment. However, in the human literature, disciplinary techniques involving ignoring or rejecting children are generally considered to be controversial (Dadds & Tully 2019) and potentially harmful for child mental health when used in the extreme (Arslan 2017). In dogs, a questionnaire-based study found that negative punishment increased the number of reported behaviour problems, but only where dogs scored highly for a ‘canine sensory processing sensitivity’ personality trait: a trait suggested to occur when dogs experience emotions strongly, making them more susceptible to stress (Bräm Dubé *et al.* 2020). This is a relatively new concept, but potentially the dogs reacting to negative punishment in the present study, may be particularly sensitive to this type of training, highlighting individuality of response. Blackwell and colleagues (2008) found that 52% of owners in their study reported using negative punishment in combination with positive punishment and/or positive reinforcement, but they found no association between training type and SRBs (Blackwell *et al.* 2008). We found no effect of positive reinforcement on SRBs in the present study, which may suggest a greater impact of aversive interactions on puppy behaviour; however most owners in the study used positive reinforcement, so we may not have had sufficient variation to detect an effect.

These results concerning negative punishment and time-outs, conflict with many SRB behavioural modification plans which often suggest ignoring the dogs or only interacting with the dog upon returning to the house when the dog is calm (Blackwell *et al.* 2006; Butler *et al.* 2011). Similarly, owners may use crates/kennels as punishment or ‘time-out’ areas (Royal Society for the Prevention of Cruelty to Animals [RSPCA] 2014). Puppies may acquire negative associations with the crate where they are meant to sleep and relax, and if the dog has problems with separation and with being enclosed, this may exacerbate their distress (Schwartz 2003; Sherman & Mills 2008; Todd 2018). Given the findings discussed earlier that experience of enclosed space at ≤ 16 weeks overnight was associated with decreased odds of SRBs at six months, it may be worth noting in preventative advice for owners to only use crates in association with positive reinforcement.

The type of positive reinforcer used may also be important, because puppies at ≤ 16 weeks who were rewarded with novel dog food treats when being trained to carry out a new behaviour had increased odds of displaying SRBs at six months of age. If this result is not a Type I error, it is unexpected and conflicts with previous research suggesting that reward-based training, including using food treats, is associated with lower numbers of owner-reported problem behaviours, compared with mixed or punishment-based training techniques (Hiby *et al.* 2004).

One explanation could be that, if owners perceive that their dogs are anxious or depressed, they may attempt to ‘cheer them up’ with novel dog treats. This could mean that provision of these rewards is a response to SRB-prone dogs, rather than a cause of SRB development. Alternatively, it is possible that there are different ‘types’ of owners: those who buy high value treats or ‘sweets’ for their dogs and those who just use normal dog food as treats. This may be linked to other owner behaviours that could influence SRBs. The link between treat-giving and problem behaviours could then be further investigated, as treat-giving is often seen by owners as being related to the dog-owner bond (Linder & Mueller 2014; White *et al.* 2016). Finally, preventing SRBs can be viewed under the general strategy of managing interactions with dogs and controlling owner attention in response to how a dog behaves. Owners who are very focused on ‘training’ with treats to specifically train behaviours may focus on this rather than on the use of their attention as an important reinforcer (Chrysafis *et al.* 2023). Only using food as a reinforcer in training may not be as effective as owner attention. In summary, the unexpected nature of this result, and the variety of possible explanations for it, means that we would not necessarily recommend that owners refrain from using novel dog treats as rewards.

### Owner responses upon returning home

We found that puppies whose owners responded to behaviour they would like to discourage when returning to their puppies at six months, by fussing over them, had significantly increased odds of displaying SRBs at six months. Fussing over puppies in response to behaviour owners would like to encourage upon owner return was not retained in the final model but was significant at univariable analysis. Consistent with our finding, some studies recommend that owners ignore their dog before departure and after returning to the household and to only initiate contact when the dog is relaxed (King *et al.* 2000; Blackwell *et al.* 2016). Owners are also often advised to avoid excessive greeting behaviour upon returning to their dog (Blackwell *et al.* 2016). McGreevy and Masters (2008) found that dogs of owners who played with them in the first 30 min of returning were more likely to display SRBs. The authors suggest the dogs may be anticipating the play behaviour upon owner return and become aroused accordingly (McGreevy & Masters 2008). Remaining calm during departure and arrival may prevent anxious responses from being reinforced at this time (Teixeira & Hall 2021) and may make coming and going generally less emotive and meaningful for the dog. Despite conflicting evidence from Teixeira and Hall (2021) who found no evidence of high arousal/excited greetings or reunions leading to increased SRBs in rescue dogs, our study appears to support the theory that calm interactions may help prevent SRBs. However, implementing this may be challenging for owners who may be inclined to greet their dog enthusiastically upon their return, meaning owner compliance to this type of advice is often poor (Blackwell *et al.* 2016).

Despite the theory, because the SRB outcome variable and the ‘fussing’ result were both at six months of age and the data are correlational, it is possible that the direction of causation may be in either direction. Owners may have been more likely to fuss over their dogs to reassure them upon return to the household, if the owners were already aware that their dogs were showing SRBs. Moreover, Konok *et al.* (2011) found that dogs who were distressed during separation tests were more likely to be ‘happy’ and show increased greeting behaviour towards their owners when reunited. Again, this means that it is difficult to make a recommendation, because avoiding fussing over puppies when greeting them may be a response to, rather than a cause of, SRBs.

### Other factors

We found that dogs belonging to younger owners (aged 16–34) had increased odds of displaying SRBs at six months compared to owners aged ≥ 35 years. While Bennett and Rohlf (2007) found conflicting results, similar findings were reported by Konok *et al.* (2015), and Brand and colleagues (2024) who found increased odds of dogs having SRBs if their owners were aged 25 to 34 years of age compared to if owners were 45 to 54 years old (Bennett & Rohlf 2007; Konok *et al.* 2015; Brand *et al.* 2024). Owner age is likely to be a proxy for an unmeasured variable (e.g. owner routine), but replication of the study would be needed to establish any further association.

We did not find significant associations between SRBs and many previously identified risk factors. For example, previous studies have suggested that mixed breed dogs were more likely to display SRBs than pure breed dogs (Flannigan & Dodman 2001). However, in the present study, breed was only significant at univariable analysis, with increased odds in mixed breed dogs and decreased odds in Labrador Retrievers (see Supplementary material) but did not contribute to the final model. Bradshaw *et al.* (2002) similarly found that SRB occurrence was not significantly associated with breed in a cross-sectional questionnaire study of dog walkers comparing SRB occurrence in mixed breed and pedigree dog breeds. Similarly, we found no effect of whether puppies attended puppy classes at ≤ 16 weeks, but rather the ways in which owners trained and interacted with their puppies seemed to influence SRB occurrence at six months. This has been previously reported (e.g. González-Martínez *et al.* 2019) but contrasts with others (Jagoe & Serpell 1996; Flannigan & Dodman 2001).

We also found no effect of maximum time left alone at six months. This factor was positively associated with SRB occurrence at 16 weeks at the univariable stage of analysis, but did not make the final model. It is recommended that owners do not leave their dogs > 4 h (Dogs Trust 2005) and previous research has suggested that dogs left for > 4 h show increased greeting behaviour, shaking and lip-licking upon owner arrival (Rehn & Keeling 2011). Interestingly, a recent paper reporting the results of a cohort study found that dogs aged 21 months of age had reduced odds of SRB if dogs had been left alone for > 4 h at 21 months compared to those who had never been left alone; however, these dogs were purchased during the 2020 pandemic and results may have been affected by owners increasing the amount of time their dogs were left alone compared to during UK lockdowns (Brand *et al.* 2024). Repetition of the present study with higher statistical power may allow the influence of both the pandemic and this variable to be investigated further as evidence on the influence of the maximum time left alone at an early age would be helpful in guiding new puppy owners on how to approach leaving their puppies.

Investigation of risk factors for SRBs in dogs older than six months may be beneficial, as research has suggested that owners tend to report canine behavioural problems at ages older than this. Indeed, one study noted that owners tended to report behavioural problems to veterinary practices when dogs were aged an average of 3.7 years (Bamberger & Houpt 2006). Furthermore, Lund and colleagues (1996) found that all behavioural problems reported in their study occurred between 18 and 33 months of age. Despite this, the present study provides evidence that signs of SRBs can occur at an early stage in a puppy’s life and emphasises the importance of effective management from an early age in order to prevent the development of these problems. Further analyses of data from the ‘Generation Pup’ cohort could investigate SRBs in these dogs at a later age to learn more about the development of SRBs over time and the long-term influence of early life factors and owner interactions.

The sample size was relatively small in this study compared to other studies (e.g. approximately 200–700: Flannigan & Dodman 2001; McGreevy & Masters 2008; van Rooy *et al.* 2018), resulting in low statistical power. Due to the multifactorial nature of separation problems in dogs it may be that there are many other factors that influence SRB occurrence at six months which were not covered in the present study. While we should be careful not to over-interpret the current findings, they can help prompt further research into the potential influence of early life experiences and owner responses towards their dogs on future development of SRBs using larger sample sizes. Future replication of the present study could also consider alternative explanations for SRBs and rule these out by considering the medical history of the dogs involved in the study (McCrave 1991). This was not conducted for the present study, but this information is available for ‘Generation Pup’ dogs whose owners have consented to permit access to their dog’s veterinary records; this could be an avenue to explore in future ‘Generation Pup’ studies. It is also important to note that due to the fact that SRBs can occur for a number of underlying reasons (e.g. frustration, fear, anxiety or boredom, see McCrave 1991; Mendl *et al.* 2010a; Burn 2017; de Assis *et al.* 2020), the group of behaviours may be considered as a syndrome (a group of behaviours which regularly occur together in a context with more than one underlying cause) meaning treatment can be challenging (de Assis *et al.* 2020). There is a range of terminology and proposed case definitions for SRBs, and it is important that each individual case has a diagnosis to inform the treatment plan for that dog.

## Animal welfare implications and conclusion

This study identified potential early-life risk factors for SRBs in dogs at six months of age. SRBs are a complicated problem in dogs which can have serious welfare implications, and early identification is important in preventing their development. It will be important that any reductions in SRB development are associated with genuine improvements to canine welfare during separation, rather than merely being reductions of the behavioural manifestations of separation-related distress.

Poor house-training progress at ≤ 16 weeks increased a puppy’s likelihood of showing problems with being left alone at six months. Providing puppies with positive experiences of being left alone and enclosed overnight from an early age, as well as encouraging sufficient sleep and rest, could help reduce the chance of puppies developing issues with being left alone at six months of age and potentially SRB that continues as the dog ages.

Many of our findings were associated with how owners responded to unfavourable dog behaviour across different contexts, but generally suggest that aversive techniques during dog-owner interactions and fussing during reunion may lead to an increased risk of SRBs at six months. We recommend that owners avoid both positive and negative punishment techniques during the general handling of puppies, as well as effusively greeting dogs upon returning to the household after separation.

Caution should be exercised in not over-interpreting these exploratory results, and efforts should be made to replicate with a larger sample. However, future research could further investigate the factors detailed here and in older dogs, and the effects of owner interactions in different scenarios and training approaches in earlier life to allow further understanding of the complicated nature of canine SRBs. We recommend replicating this study, with the addition of video footage (Palestrini *et al.* 2010) to potentially see the effect of time left alone, which would additionally allow subtler SRBs, such as pacing, spinning and trembling, to be detected with greater certainty than in questionnaire-based studies. Furthermore, repetition of the study could further investigate the potential influence of puppy provenance and the pandemic on SRBs in the long-term. These results are promising and may ultimately improve animal welfare and contribute to positive change regarding how owners approach responding to puppy ‘problem behaviours’, especially as the results reported here indicate that owner interactions may play a part in this behavioural problem.

## Supporting information

Dale et al. supplementary material Dale et al. supplementary material

## Supplementary material

The supplementary material for this article can be found at http://doi.org/10.1017/awf.2024.56.
