# Supplementary material for: Canine separation-related behaviour at six months of age: Dog, owner and early-life risk factors identified using the ‘Generation Pup’ longitudinal study
Source: Anim Welf. 2024 Dec 16;33:e60. doi: 10.1017/awf.2024.56 (PMC11655275; doi:10.1017/awf.2024.56)
Supplement: Dale et al. supplementary material [file S0962728624000563sup001.pdf]

Canine separation-related behaviour at six months of age: Dog, owner and early-life risk factors identified using the 'Generation Pup' longitudinal study

Fiona C Dalehttps://orcid.org/0009-0006-3948-7938, Charlotte C Burn, Jane Murray, and Rachel Casey

Table S1. Recategorisation of categories of questions from the Generation Pup questionnaires for univariate and multivariate statistical analysis (variables which reached *P* < 0.200 shown)

Variable name/question	Questionnaire/ Time-point	Original categories in Generation Pup	New Variable Name	Categories used for analysis
**My puppy is a/an**	About My Puppy	List of dog breeds presented to owners	Dog Breed	• Crossbreeds<br>• Labrador Retriever<br>• Other pure breeds
**Derived Kennel Club classification from breed names**	About My Puppy	None and Unknown<br>Working<br>Pastoral<br>Hound<br>Terrier<br>Utility<br>Toy<br>Gundog	Kennel Club Group	• None and Unknown<br>• Working, Hound, Terrier, Utility and Toy<br>• Pastoral<br>• Gundog
**I am aged…**	About Me	16-24 years<br>25-34 years<br>35-44 years<br>45-54 years<br>55-64 years<br>65 years and older<br>Prefer not to state	Owner Age	• 16-34 years<br>• 35 years and older

**My marital status is...**	About Me	Single<br>Common law partnered/Cohabiting<br>Married<br>Civil partnered<br>Divorced/Separated<br>Widowed<br>Prefer not to state	Marital Status Combined	• Single & Widowed<br>• Common Law<br>• Married & Civil Partnered<br>• Divorced/Separated
**I live in a…**	About My Household	City or urban area<br>Suburban area<br>Village or small town<br>Rural area (such as small village or hamlet)<br>In a remote/isolated area<br>Other	Home Location Combination	• City or urban area<br>• Suburban area<br>• Village or small town or<br>• Rural or remote
**The longest period that my puppy has been left at home without human company (during the day/evening or night) was...**	≤16 weeks	Less than 1 hour<br>One or more hours but less than three hours<br>Three or more hours but less than six hours<br>Six or more hours<br>I don't know	Maximum Period Left at ≤16 weeks combination	• Not Left Alone<br>• Less than 1 hour<br>• One or more hours but less than three hours<br>• Three or more hours but less than six hours and<br>• Six or more hours
**The type of enclosed space used for my puppy was…**	≤16 weeks	A crate or indoor kennel<br>A utility room or kitchen area which may be shut off from the rest of the house<br>A garage/out-building	Type of Enclosed Space Combination at ≤16 weeks	• Not enclosed<br>• Any crate experience<br>• Other (e.g. utility room or kitchen area only, play pen, area of house sectioned off by gate etc)

		An outdoor kennel<br>Other (please specify)		
**I have used an enclosed space for my puppy…**	≤16 weeks	To make sure he/she had a safe space<br>To separate him/her from other household pets<br>To keep him/her calm/quiet<br>To keep him/her still if he/she has been naughty<br>To house train him/her<br>Other (please specify)	Reason Enclosed at ≤16 weeks	• Not enclosed<br>• Calm and/or safe only (plus sleep only) and house train<br>• Different combinations but include separate from others or keep still if naughty
**My puppy goes in the enclosed space…**	≤16 weeks	Occasionally<br>Regularly, in the day, for less than 3 hours total<br>Regularly, in the day, for 3 or more hours in total<br>Overnight	Frequency Enclosed at ≤16 weeks day/night	• Not enclosed<br>• Day Only (not mention overnight)<br>• Combinations of time but experience of being enclosed overnight
**My puppy sleeps during the night-time for a total of approximately ….**	≤16 weeks	Owner entered maximum number of hours	Maximum Hours Sleep at Night at ≤16 weeks	• 6 hours to 8 hours<br>• 9 hours<br>• 10 hours and over<br>• Unspecified
**My puppy sleeps during the night-time for a total of approximately ….**	≤16 weeks	Owner entered minimum number of hours	Minimum Hours Sleep at Night at ≤16 weeks	• 0 hours - 4 hours<br>• 5 hours<br>• 6 hours<br>• 7 hours<br>• 8 hours or more<br>• Unspecified
**My puppy sleeps during the day-time for a total of**	≤16 weeks	Owner entered maximum number of hours	Maximum Hours Sleep During the Day at ≤16 weeks	• 2 hours - 3 hours<br>• 4 hours<br>• 5 hours<br>• 6 hours<br>• 7 hours and over

**approximately ….**				• Unspecified
**At the moment, my puppy is settled to sleep at night…**	≤16 weeks	By having him/her in a bedroom with human company<br>By sleeping in another room in the house with him/her (for example sleeping in the living room)<br>By waiting for him/her to be asleep before going to bed in a different room<br>By leaving him/her in a room/area without human company<br>By leaving him/her in an outbuilding (for example kennel, stable)	Method of settling at ≤16 weeks combination	• Sleeping in room<br>• (By having him/her in a bedroom with human company<br>• By sleeping in another room in the house with him/her (for example sleeping in the living room)<br>• By waiting for him/her to be asleep before going to bed in a different room)<br>• Leaving Alone<br>• (By leaving him/her in a room/area without human company<br>• By leaving him/her in an outbuilding (for example kennel, stable)
**At night my puppy sleeps…**	≤16 weeks	In a kennel/crate<br>On a dog bed<br>On a chair/other furniture<br>On a person's bed<br>On the floor<br>Other (please specify)	Sleep Location at ≤16 weeks combination	• Anything not including kennel/crate or person's bed (on a dog bed, on a chair/furniture, on the floor)<br>• Kennel/crate plus other locations<br>• Kennel/crate only<br>• Person's bed mentioned (with/without other locations)
**My puppy's housetraining…**	≤16 weeks	Is complete, my puppy no longer has	Housetraining Progression	• Zero to three accidents a week

		any accidents indoors<br>Is ongoing, and there are fewer than four accidents a week<br>Is ongoing, but there are still four or more accidents per week	at ≤16 weeks combination	• Is ongoing, but there are still four or more accidents per week
**I am training, or want to train my puppy to toilet by…**	≤16 weeks	Taking/letting him/her outside regularly<br>Telling him/her off when he/she toilets inside<br>Praising him/her when he/she toilets in the correct place<br>Using puppy pads/newspapers on the floor to show areas where he/she may toilet<br>I haven't started this training yet<br>Other (please specify)	House Train Method at ≤16 weeks combination	• Taken out regularly and/or praised<br>• Telling off mentioned and/or praise not mentioned
**At the party/class, I was given information or shown how to…**	≤16 weeks	Given information about this, but NOT shown how to do this<br>Shown how to do this, but NOT given separate information about this	Puppy Training Class House Training Info at ≤16 weeks Combination	• No classes<br>• Given information about this, but NOT shown how to do this<br>• Shown how to do this, AND/OR given separate information about this<br>• This was not covered

		Shown how to do this AND given separate information about this<br>This was not covered		
**When training my puppy to do a new behaviour, I use the following rewards…**	≤16 weeks	Verbal praise (for example 'good boy/girl')<br>Physical praise (for example pat/stroke/cuddle)<br>A toy<br>A ball<br>A clicker<br>Some of his/her normal dog food<br>A dog food other than his/her own kibble/biscuit<br>Dog food treats<br>Human food (for example cheese/sausage\)<br>None/no other rewards are used<br>Training not started<br>Other (please specify)	Training Reward – Kibble or dog food treat at ≤16 weeks	• No use of kibble and/or dog food treats<br>• Kibble and/or dog food treats
**When correcting my puppy for doing something wrong, I would…**	≤16 weeks	Tell him/her off<br>Use a physical reprimand/correction (for example smack or tap him/her on	Correction Method at ≤16 weeks combination	• Move away, withhold treats, ignore, don't use anything, other(e.g. Show him/her correct behaviour, redirect behaviour)<br>• Tell off, physical, distraction,

		nose or bottom)<br>Distraction (for example rattle can/loud noise/water spray)<br>Withold treats<br>Ignore him/her<br>Use physical manipulation (for example putting him/her into a sit or pushing him/her if he/she jumps up)<br>Hold him/her still<br>I don't use anything<br>Other (please specify)		manipulation, hold still (may use response categories too (e.g. move away, ignore etc)
**In total, the number of nights my puppy has been separated from me, and all household members (if applicable) since he/she came to live me is…**	6 months	Not stayed away<br>One night<br>2-5 nights<br>6-10 nights<br>11-15 nights<br>More than 15 nights<br>I don't know/can't remember	Nights separated at six-months combination	• Not stayed away<br>• One to five nights<br>• 6-10 nights<br>• 11 or more nights
**In general, during the last seven days, when I/we were relaxing at home with my puppy and he/she behaved in a way that I/we would like**	6 months	Verbally praise him/her (for example saying 'good dog')<br>Play with him/her<br>Give him/her a food treat	Response to Good Behaviour when Relaxing at six-months	• No cuddle or stroke<br>• Cuddle and/or stroke

**him/her to, I/we would…**		Ignore him/her<br>Stroke him/her<br>Cuddle him/her<br>Not applicable, he/she has not generally behaved in a way I would like to encourage<br>Other (please specify)		
**In general, during the last seven days, when I/we were relaxing at home with my puppy and he/she behaved in a way that I/we would NOT like him/her to, I/we would…**	6 months	Tell him/her off<br>Use a physical reprimand/correction (for example smack or tap him/her on nose or bottom)<br>Distract him/her (for example rattle can/loud noise/water spray)<br>Move away from him/her<br>Don’t give him/her treats that would normally be given at this time<br>Ignore him/her<br>Physically manipulate him/her (for example, hold still, put him/her into a sit or push	Response to Bad Behaviour when Relaxing at six-months	• ≤1 aversive techniques and no timeouts<br>• ≤1 aversive techniques but time-outs<br>• ≥2 aversive techniques

		off if he/she jumps up)<br>Play with him/her<br>Stroke him/her<br>Cuddle him/her<br>Hold him/her still<br>Encourage him/her to calm down<br>Let him/her outside into the garden or yard<br>Move him/her to another part of the house<br>Not applicable, he/she has not generally behaved ina way I would like to discourage<br>Other (please specify)		
**In general, during the last seven days, when I was preparing to leave my puppy behind in the house (without human company), and she/he behaved in a way that I/we would like him/her to, I/we would…**	6 months	Verbally praise him/her (for example saying 'good dog')<br>Play with him/her<br>Give him/her a food treat<br>Ignore him/her<br>Stroke him/her<br>Cuddle him/her<br>Not applicable, he/she has not generally	Response to Good Behaviour when Preparing to Leave Without Puppy at six-months	• No stroke and/or cuddle<br>• Stroke and/or cuddle mentioned

		behaved in a way I would like to encourage<br>Other (please specify)		
**In general, during the last seven days, when I was preparing to leave my puppy behind in the house (without human company), and she/he behaved in a way that I/we would NOT like him/her to, I/we would…**	6 months	Tell him/her off<br>Use a physical reprimand/correction (for example smack or tap him/her on nose or bottom)<br>Distract him/her (for example rattle can/loud noise/water spray)<br>Move away from him/her<br>Don't give him/her treats that would normally be given at this time<br>Ignore him/her<br>Physically manipulate him/her (for example, hold still, put him/her into a sit or push off if he/she jumps up)<br>Play with him/her<br>Stroke him/her<br>Cuddle him/her<br>Hold him/her still	Response to Bad Behaviour when Preparing to Leave without Puppy at six-months	• ≤1 aversive technique<br>• ≥2 aversive techniques<br>• Not applicable mentioned

		Encourage him/her to calm down<br>Let him/her outside into the garden or yard<br>Move him/her to another part of the house<br>Give him/her a food treat<br>Not applicable, he/she has not generally behaved ina way I would like to discourage<br>Other (please specify)		
**In general, during the last seven days, when I was preparing to leave the house WITH my puppy (for example putting on coat, picking up keys, picking up lead or harness), and my puppy knew he/she was coming too and he/she behaved in a way that I/we would NOT like him/her to, I/we would…**	6 months	Tell him/her off<br>Use a physical reprimand/correction (for example smack or tap him/her on nose or bottom)<br>Distract him/her (for example rattle can/loud noise/water spray)<br>Move away from him/her<br>Don't give him/her treats that would normally be given at this time<br>Ignore him/her	Response to Bad Behaviour when Preparing to Leave with Puppy at six-months	• No aversive techniques*<br>• One or more aversive techniques

		Physically manipulate him/her (for example, hold still, put him/her into a sit or push off if he/she jumps up)<br>Play with him/her<br>Stroke him/her<br>Cuddle him/her<br>Hold him/her still<br>Encourage him/her to calm down<br>Let him/her outside into the garden or yard<br>Move him/her to another part of the house<br>Give him/her a food treat<br>Not applicable, he/she has not generally behaved in a way I would like to discourage<br>Other (please specify)		
**During the last seven days, when left at home without human company my puppy was usually left…**	6 months	Confined to his/her outside kennel<br>Confined to his/her indoor kennel<br>Outside, with free range of the garden	Left Alone Access at six-months	• Confined to his/her indoor crate/outdoor kennel<br>• Restricted to one room/area of the home<br>• With free range of house OR in the house with access to the garden/OR

		Restricted to one room/area of the home<br>With free range of the house<br>In the house with access to garden<br>Other (please specify)		outside, with free range of the garden
**In general, during the last seven days, my puppy has slept…**	6 months	Indoors, in a bedroom with human company<br>Indoors, in a room without human company<br>Outdoors, in an outbuilding (for example stable or barn)<br>Outdoors, ina kennel or small sheltered area<br>Other (please specify)	Sleeping Location Type at six-months	• Indoors, in a bedroom with human company (With human company)<br>• Indoors, in a room without human company/Outdoors, in a kennel or small sheltered area (Without human company)
**In general, during the last seven days, my puppy has slept…**	6 months	On the floor<br>On furniture (other than a bed)<br>On a human bed<br>In a crate/kennel<br>On a dog bed<br>I don't know<br>Other (please specify)	Normal Sleeping Location at six-months	• Only dog bed, floor, other<br>• Mention crate (no human bed or furniture)<br>• Mention human bed and/or furniture
**In general, during the last seven days, when I or other members of the**	6 months	Verbally praise him/her (for example	Response to Good Behaviour when No	• No stroke and/or cuddle<br>• Stroke and/or cuddle

**household were not paying attention to my puppy, and he/she behaved in a way that I/we would like him/her to, I/we would…**		saying 'good dog')<br>Play with him/her<br>Give him/her a food treat<br>Ignore him/her<br>Stroke him/her<br>Cuddle him/her<br>Not applicable, he/she has not generally behaved in a way I would like to encourage<br>Other (please specify)	Attention at six-months	
**In general, during the last seven days, when I or other members of the household were not paying attention to my puppy, and he/she behaved in a way that I/we would NOT like him/her to, I/we would…**	6 months	Tell him/her off<br>Use a physical reprimand/correction (for example smack or tap him/her on nose or bottom)<br>Distract him/her (for example rattle can/loud noise/water spray)<br>Move away from him/her<br>Don't give him/her treats that would normally be given at this time<br>Ignore him/her	Response to Bad Behaviour when No Attention at six-months	• ≤1 aversive techniques<br>• ≥2 aversive techniques<br>• Not applicable mentioned

		Physically manipulate him/her (for example, hold still, put him/her into a sit or push off if he/she jumps up)<br>Play with him/her<br>Stroke him/her<br>Cuddle him/her<br>Hold him/her still<br>Encourage him/her to calm down<br>Let him/her outside into the garden or yard<br>Move him/her to another part of the house<br>Give him/her a food treat<br>Not applicable, he/she has not generally behaved in a way I would like to discourage<br>Other (please specify)		
**In general, during the last seven days, when my puppy was separated (for example by a closed or baby gate) from myself or another member of the**	6 months	Tell him/her off<br>Use a physical reprimand/correction (for example smack or tap him/her on nose or bottom)	Response to Bad Behaviour when Separated at Home at six-months	• ≤1 aversive techniques<br>• ≥2 aversive techniques<br>• Not applicable

**household while in the home, and he/she behaved in a way that I/we would NOT like him/her to, I/we would…**	Distract him/her (for example rattle can/loud noise/water spray)<br>Move away from him/her<br>Don’t give him/her treats that would normally be given at this time<br>Ignore him/her<br>Physically manipulate him/her (for example, hold still, put him/her into a sit or push off if he/she jumps up)<br>Play with him/her<br>Stroke him/her<br>Cuddle him/her<br>Hold him/her still<br>Encourage him/her to calm down<br>Let him/her outside into the garden or yard<br>Move him/her to another part of the house<br>Give him/her a food treat<br>Not applicable, he/she has

		not generally behaved in a way I would like to discourage<br>Other (please specify)		
**In general, during the last seven days, when my puppy had been left at home WITHOUT human company and I/we returned to the house, and he/she behaved in a way that I/we would NOT like him/her to, I/we would…**	6 months	Tell him/her off<br>Use a physical reprimand/correction (for example smack or tap him/her on nose or bottom)<br>Distract him/her (for example rattle can/loud noise/water spray)<br>Move away from him/her<br>Don’t give him/her treats that would normally be given at this time<br>Ignore him/her<br>Physically manipulate him/her (for example, hold still, put him/her into a sit or push off if he/she jumps up)<br>Play with him/her<br>Stroke him/her<br>Cuddle him/her	Response to Bad Behaviour upon Return at six-months	• ≤1 aversive techniques<br>• ≥2 aversive techniques<br>• Not applicable
			Response to Bad Behaviour upon Return at six-months - Greet/Tell Off	• No greet or tell off<br>• Greet and/or tell off<br>• No greet or tell off but not applicable
			Response to Bad Behaviour upon Return at six-months – ‘Fuss’	• No play/stroke/cuddle/greet<br>• Play/stroke/cuddle/greet<br>• Not applicable

	Hold him/her still<br>Encourage him/her to calm down<br>Let him/her outside into the garden or yard<br>Move him/her to another part of the house<br>Give him/her a food treat<br>Not applicable, he/she has not generally behaved in a way I would like to discourage<br>Other (please specify)		
**In the last two months I have taken my puppy to a veterinary practice**	Yes<br>No, but a vet visited my home instead/there is a vet in the household<br>Not at all, my puppy has not seen a vet in the last two months	Vet Visit at six-months	• A visit of any type (Yes or No, but a vet visited my home instead/there is a vet in the household)<br>• Not at all, my puppy has not seen a vet in the last two months

* Aversive techniques: Tell him/her off, physically reprimand/correct him/her (for example smack or tap on nose or bottom), distract him/her (for example with a rattled can/loud noise/water spray), physically manipulate him/her (for example hold still, put into a sit, or push off if he/she jumps up), hold him/her still

Table S2. Variables that reached threshold significance of *P* ≤ 0.200 in univariable analysis for risk factors (n = 145).

Variable name	Categories	Reference Category	*P*-value	OR	CI

**Dog Breed (Combined categories)**	Crossbreeds	Unknown/known breeds/crosses	0.020		
	Labrador Retriever		0.005	0.172	0.05-0.594
	Other pure breeds		0.188	0.618	0.303-1.264
**Kennel Club Group (Combined Categories)**	None and Unknown	None and Unknown	0.001		
	Working, Hound, Terrier, Utility and Toy		0.900	1.055	0.457-2.433
	Pastoral		0.073	0.371	0.126-1.095
	Gundog		0.001	0.218	0.092-0.517
**Pure v Cross Breed**	Pure breed	Pure breed			
	Cross breed		0.057	1.967	0.980-3.949
**Owner Age**	16-34 years	16-34 years			
	35 years and older		<0.001	5.349	2.025-14.126
**Marital Status Combo**	Single & Widowed	Single & Widowed	0.045		
	Common Law		0.395	1.76	0.479-6.467
	Married & Civil Partnered		0.131	0.512	0.215-1.22
	Divorced/Separated		0.081	0.21	0.036-1.21
**Home Location Combo Plus**	City or urban area	City or urban area	0.198		
	Suburban area		0.072	0.395	0.144-1.086
	Village or small town or Rural or remote		0.191	0.549	0.224-1.348
**Maximum Period Left at ≤16 weeks**	Not Left Alone	Not Left Alone	0.063		
	Less than 1 hour and One or more hours but less than three hours		0.435	1.5	0.542-4.155
	Three or more hours but less than six hours and Six or more hours		0.042	2.946	1.042-8.335
**Type of Enclosed Space Combo at ≤16 weeks**	Not enclosed	Not enclosed	<0.001		
	Any crate experience		<0.001	0.176	0.069-0.45
	Other		0.420	0.583	0.158-2.159
**Reason Enclosed at ≤16 weeks**	Not enclosed	Not enclosed	0.003		
	Calm and/or safe only (plus sleep only) and housetrain		0.001	0.195	0.076-0.505
	Diff combinations but include separate from		0.018	0.259	0.084-0.794

	others or keep still if naughty				
**Frequency Enclosed at ≤16 weeks**	Not enclosed	Not enclosed	<0.001		
	Day Only (not mention overnight)		0.093	0.407	0.142-1.163
	Combinations of time but experience of being enclosed overnight		<0.001	0.139	0.052-0.369
**Maximum Hours Sleep at Night at ≤16 weeks**	6 hours to 8hours	6 hours to 8 hours	0.018		
	9 hours		0.036	0.375	0.15-0.94
	10 hours and over		0.007	0.161	0.043-0.605
	Unspecified		0.415	0.7	0.297-1.649
**Minimum Hours Sleep at Night at ≤16 weeks**	0 hours - 4 hours	0 hours - 4 hours	0.068		
	5 hours		0.180	2.554	0.648-10.059
	6 hours		0.911	1.061	0.375-3.006
	7 hours		0.275	0.546	0.184-1.619
	8 hours or more		0.042	0.273	0.078-0.954
	Unspecified		0.789	0.867	0.304-2.474
**Maximum Hours Sleep During the Day at ≤16 weeks**	2 hours - 3 hours	2 hours - 3 hours	0.116		
	4 hours		0.184	0.457	0.144-1.449
	5 hours		0.073	0.286	0.073-1.124
	6 hours		0.158	0.39	0.105-1.442
	7 hours and over		0.954	0.964	0.283-3.284
	Unspecified		0.747	1.169	0.453-3.014
**Method of settling at ≤16 weeks**	Sleeping in room (By having him/her in a bedroom with human company OR By sleeping in another room in the house with him/her (for example sleeping in the living room) OR By waiting for him/her to be asleep before going to bed in a different room)	Sleeping in room			

	Leaving Alone<br>(By leaving him/her in a room/area without human company OR<br>By leaving him/her in an outbuilding (for example kennel, stable))		0.021	0.45	0.228-0.889
**Access to People at ≤16 weeks**	No<br>Yes	No	 0.008	 3.136	 1.343-7.324
**Sleep Location at ≤16 weeks**	Anything not including kennel<br>Kennel plus something<br>Kennel only<br>Persons bed at all	Anything not including kennel	0.001<br>0.340<br>0.002<br>0.330	 0.591<br>0.273<br>1.92	 0.2-1.742<br>0.118-0.631<br>0.516-7.144
**Housetraining Progression at ≤16 weeks**	Zero to three accidents per week<br>Is ongoing, but there are still four or more accidents per week	Zero to three accidents per week	 <br><0.001	 <br>4.356	 <br>1.861-10.197
**House Train Method at ≤16 weeks**	Taken out regularly and/or praised<br>Telling off mentioned and/or praise not mentioned	Taken out regularly and/or praised	 <br>0.107	 <br>2.164	 <br>0.846-5.531
**Puppy Training Class House Training Info at ≤16 weeks**	No classes<br>Given information about this, but NOT shown how to do this Shown how to do this, AND/OR given separate information about this<br>This was not covered	No classes	0.060<br>0.810<br>0.103<br>0.116	 1.122<br>2.848<br>0.534	 0.438-2.875<br>0.811-10.008<br>0.244-1.167
**Puppy Class Run by Vet Practice at ≤16 weeks**	No classes<br>No<br>Yes	No classes	0.082<br>0.029<br>0.273	 3.327<br>1.483	 1.132-9.779<br>0.733-2.999
**Training Reward – Kibble or dog food treat at ≤16 weeks**	No use of kibble and/or dog food treats<br>Kibble and/or dog food treats	No use of kibble and/or dog food treats	 <br>0.003	 <br>3.529	 <br>1.521-8.189

**Correction Method at ≤16 weeks**	Move away, withhold treats, ignore, don't use anything, other<br>Tell off, physical, distraction, manipulation, hold still (may use others too)	Move away, withhold treats, ignore, don't use anything, other	0.010	2.591	1.251-5.367
**Experience Gunshot at six-months**	Definitely No<br>Definitely Yes<br>I don't know	Definitely No	0.105<br>0.049<br>0.261	0.479<br>0.514	0.230-0.996<br>0.161-1.639
**Experience Raised Voices at six-months**	Definitely No<br>Definitely Yes	Definitely No	0.043	1.935	1.022-3.667
**Nights Separated at six-months**	Not stayed away<br>One to five nights<br>6-10 nights<br>11 or more nights	Not stayed away	0.106<br>0.475<br>0.086<br>0.131	1.325<br>2.981<br>0.294	0.613-2.866<br>0.856-10.377<br>0.06-1.438
**Response to Good Behaviour when Relaxing at six-months**	No cuddle or stroke<br>Cuddle and/or stroke	No cuddle or stroke	0.062	3.033	0.944-9.744
**Response to Bad Behaviour when Relaxing at six-months**	≤1 aversive techniques<br>≤1 aversive techniques but time-outs<br>≥2 aversive techniques	≤1 aversive techniques	0.039<br>0.018<br>0.018	3<br>3.25	1.205-7.47<br>1.227-8.605
**Response to Good Behaviour when Preparing to Leave Without Puppy at six-months (Includes 4 NAs)**	No stroke and/or cuddle<br>Stroke and/or cuddle mentioned	No stroke and/or cuddle	0.010	2.337	1.228-4.448
**Response to Bad Behaviour when Preparing to**	≤1 aversive technique<br>≥2 aversive techniques<br>Not applicable mentioned	≤1 aversive technique	0.002<br>0.039<br>0.021	3.5<br>0.442	1.062-11.534<br>0.221-0.883

**Leave without Puppy at six-months**					
**Response to Bad Behaviour when Preparing to Leave with Puppy at six-months**	No aversive techniques	≤1 aversive technique			
	One or more aversive techniques		0.043	1.927	1.02-3.643
**Left Alone Access at six-months**	Confined to his/her indoor crate/outdoor kennel	Confined to his/her indoor crate/outdoor kennel	0.048		
	Restricted to one room/area of the home		0.018	2.31	1.156-4.615
	With free range of house OR in the house with access to the garden/OR outside, with free range of the garden		0.117	2.117	0.829-5.409
**Sleeping Location Type at six-months**	Indoors, in a bedroom with human company (With human company)	Indoors, in a bedroom with human company (With human company)			
	Indoors, in a room without human company/Outdoors, in a kennel or small sheltered area (Without human company)		0.013	0.432	0.223-0.837
**Normal Sleeping Location at six-months**	Only dog bed, floor, other	Only dog bed, floor, other	0.001		
	Mention crate (no human bed or furniture)		0.951	1.027	0.442-2.383
	Mention human bed and/or furniture		0.004	3.896	1.551-9.783
**Access People at Night at six-months**	No	No			
	Yes		0.148	1.624	0.841-3.135
**Response to Good Behaviour when No Attention at six-months**	No stroke and/or cuddle	No stroke and/or cuddle			
	Stroke and/or cuddle		0.04	2.19	1.036-4.627
**Response to Bad**	≤1 aversive techniques	≤1 aversive techniques	0.005		
	≥2 aversive techniques		0.003	3.516	

**Behaviour when No Attention at six-months**	Not applicable mentioned		0.446	0.684	1.528-8.091<br>0.257-1.817
**Response to Bad Behaviour when Separated at Home at six-months**	≤1 aversive techniques<br>≥2 aversive techniques<br>Not applicable	≤1 aversive techniques	0.015<br>0.111<br>0.033	 2.708<br>0.44	 0.796-9.211<br>0.207-0.936
**Puppy Behavioural Problem at six-months**	No<br>Yes	No	 0.013	 2.553	 1.220-5.341
**Response to Good Behaviour upon Return at six-months**	No cuddle mentioned<br>Cuddle mentioned	No cuddle mentioned	 0.002	 2.826	 1.458-5.476
**Response to Bad Behaviour upon Return at six-months**	≤1 aversive techniques<br>≥2 aversive techniques<br>Not applicable	≤1 aversive techniques	0.003<br><br>0.052<br><br>0.011	 <br>8.143<br><br>0.425	 <br>0.985-67.303<br><br>0.219-0.823
**Response to Bad Behaviour upon Return at six-months - Greet/Tell Off**	No greet or tell off<br>Greet and/or tell off<br>No greet or tell off but NA	No greet or tell off	0.001<br>0.033<br>0.171	 2.485<br>0.571	 1.074-5.748<br>0.256-1.275
**Response to Bad Behaviour upon Return at six-months – 'Fuss'**	No play/stroke/cuddle/greet<br>Play/stroke/cuddle/greet<br>Not applicable	No play/stroke/cuddle/greet	0.001<br>0.063<br>0.053	 2.237<br>0.481	 0.956-5.234<br>0.229-1.01
**Showing Teething at six-months**	No<br>Yes	No	 0.094	 1.771	 0.908-3.457
**Vet Visit at six-months**	Some visit of any type (Yes or No, but a vet visited my home instead/there is a vet in the household)	Yes	0.121	0.564	0.273-1.164

	Not at all, my puppy has not seen a vet in the last two months	
